# The G51D *SNCA* mutation generates a slowly progressive α-synuclein strain in early-onset Parkinson’s disease

**DOI:** 10.1186/s40478-023-01570-5

**Published:** 2023-05-03

**Authors:** Heather H. C. Lau, Ivan Martinez-Valbuena, Raphaella W. L. So, Surabhi Mehra, Nicholas R. G. Silver, Alison Mao, Erica Stuart, Cian Schmitt-Ulms, Bradley T. Hyman, Martin Ingelsson, Gabor G. Kovacs, Joel C. Watts

**Affiliations:** 1 Tanz Centre for Research in Neurodegenerative Diseases, University of Toronto, Krembil Discovery Tower, Rm. 4KD481, 60 Leonard Ave., Toronto, ON M5T 0S8 Canada; 2 Department of Biochemistry, University of Toronto, Toronto, ON Canada; 3 Department of Neurology, Massachusetts General Hospital, Charlestown, MA USA; 4 Department of Radiology, Massachusetts General Hospital, Charlestown, MA USA; 5 Neuroscience Program, Harvard Medical School, Boston, MA USA; 6 Department of Public Health and Caring Sciences/Geriatrics, Uppsala University, Uppsala, Sweden; 7 Krembil Brain Institute, University Health Network, Toronto, ON Canada; 8 Department of Medicine, University of Toronto, Toronto, ON Canada; 9 Department of Laboratory Medicine and Pathobiology, University of Toronto, Toronto, ON Canada

**Keywords:** Parkinson’s disease, Multiple system atrophy, α-synuclein, Protein aggregation, Strain, Propagation, Transgenic mice, Neuropathology, Seed amplification assay

## Abstract

Unique strains of α-synuclein aggregates have been postulated to underlie the spectrum of clinical and pathological presentations seen across the synucleinopathies. Whereas multiple system atrophy (MSA) is associated with a predominance of oligodendroglial α-synuclein inclusions, α-synuclein aggregates in Parkinson’s disease (PD) preferentially accumulate in neurons. The G51D mutation in the *SNCA* gene encoding α-synuclein causes an aggressive, early-onset form of PD that exhibits clinical and neuropathological traits reminiscent of both PD and MSA. To assess the strain characteristics of G51D PD α-synuclein aggregates, we performed propagation studies in M83 transgenic mice by intracerebrally inoculating patient brain extracts. The properties of the induced α-synuclein aggregates in the brains of injected mice were examined using immunohistochemistry, a conformational stability assay, and by performing α-synuclein seed amplification assays. Unlike MSA-injected mice, which developed a progressive motor phenotype, G51D PD-inoculated animals remained free of overt neurological illness for up to 18 months post-inoculation. However, a subclinical synucleinopathy was present in G51D PD-inoculated mice, characterized by the accumulation of α-synuclein aggregates in restricted regions of the brain. The induced α-synuclein aggregates in G51D PD-injected mice exhibited distinct properties in a seed amplification assay and were much more stable than those present in mice injected with MSA extract, which mirrored the differences observed between human MSA and G51D PD brain samples. These results suggest that the G51D *SNCA* mutation specifies the formation of a slowly propagating α-synuclein strain that more closely resembles α-synuclein aggregates associated with PD than MSA.

**Supplementary Information:**

The online version contains supplementary material available at 10.1186/s40478-023-01570-5.

## Introduction

The synucleinopathies are a group of progressive neurodegenerative disorders that include Parkinson’s disease (PD) as well as multiple system atrophy (MSA) and dementia with Lewy bodies (DLB). These disorders derive their name from the presence of extensive intracellular inclusions of aggregated α-synuclein (α-syn) protein in patient brains [23]. Mutation or multiplication of the *SNCA* gene, which encodes α-syn, causes familial forms of the synucleinopathies, pointing to a central role for α-syn in disease pathogenesis [10, 50, 64]. The cellular tropism of α-syn aggregates differs markedly among the synucleinopathies. For instance, MSA is characterized by a predominance of glial cytoplasmic inclusions (GCIs), deposits of aggregated and misfolded α-syn protein in oligodendrocytes, whereas α-syn-containing Lewy bodies (LBs) and Lewy neurites within neurons are the pathological signature of PD and DLB [67, 73, 74, 79]. MSA patients also display a more aggressive progression of motor symptoms as well as autonomic dysfunction, providing a clinical distinction to PD [16]. α-Synuclein is a 140-amino acid pre-synaptic protein that exists physiologically as either an intrinsically disordered monomer or an α-helical tetramer [3, 18]. Polymerization of α-syn into β-sheet-rich aggregates, such as fibrils, is thought to contribute to disease pathogenesis in the synucleinopathies. Evidence across cell culture and animal models has shown that pathological α-syn aggregates exhibit prion-like properties [30]. Like prions, α-syn aggregates can act as seeds that template the misfolding of normal α-syn into the disease-associated state. For example, application of in vitro-generated α-syn pre-formed fibrils (PFFs) to cultured cells or injection of PFFs into mice results in the induction and propagation of α-syn pathology [41–43, 77]. The ability of α-syn aggregates to spread from cell-to-cell within regions of the brain and from the periphery to the central nervous system provides a potential molecular explanation for the hierarchical pattern of Lewy pathology progression observed in PD [7, 9, 33, 52, 75].

Converging evidence implies that distinct strains of α-syn aggregates underlie the clinical and pathological heterogeneity among the synucleinopathies [28, 65]. Protein aggregate strains, which were first described in the prion diseases, are structurally distinct assemblies that cause unique disease phenotypes [66]. Like the prion diseases, the synucleinopathies are heterogeneous in their progression rates, symptoms, affected brain regions and the cell types susceptible to developing protein inclusions [23]. Recombinant α-syn can polymerize into multiple types of conformationally distinct aggregates that elicit unique pathological phenotypes upon injection into animal models [6, 12, 26, 34, 48]. Moreover, compelling data have been obtained arguing that the α-syn aggregates present in PD and MSA constitute conformationally-distinct strains [49, 51, 62, 76, 85]. α-Syn aggregates purified from the brains of patients with either MSA or Lewy body pathology have distinct filament structures [60, 86], and α-syn aggregates from MSA patients propagate more rapidly and exhibit much higher seeding activity in cultured cell and animal bioassays relative to PD- and DLB-derived aggregates [2, 36, 40, 45, 49, 51, 71, 80–82, 84]. It has been proposed that the enhanced propagation properties of MSA α-syn aggregates may be related to factors found within the cellular environments of oligodendrocytes versus neurons [19, 49].

In prion diseases, mutations within the prion protein can lead to the formation of distinct prion strains [70]. Whether the same is true of α-syn mutations within the synucleinopathies remains unknown. The G51D α-syn mutation has been found in four different cohorts of familial PD [31, 32, 39, 72]. This mutation is of particular interest due to the early disease onset and rapid progression as well as overlapping clinical and pathological indicators of MSA. Of note, G51D PD patients display autonomic disturbances and, in addition to prominent Lewy pathology, α-syn-containing GCIs within the brain [31, 32]. Studies on G51D-mutant recombinant α-syn have revealed an equal or lowered aggregation propensity [17, 20, 39, 59] alongside impaired membrane binding ability [55, 68], increased secretion [25], and higher β-sheet content once polymerized into aggregates [27] relative to wild-type α-syn. Transmission studies involving the intracerebral inoculation of PFFs into rodent models have shown that G51D-mutant α-syn can template the misfolding and spread of aggregated α-syn in the recipient brain [25, 27, 58].

Here, we have investigated the transmission properties of α-syn aggregates from two cases of G51D PD using M83 transgenic mice (TgM83) [22]. While MSA aggregates induced robust disease, animals inoculated with G51D PD exhibited a very slowly progressive, subclinical synucleinopathy with a neuropathological signature distinct from MSA-inoculated mice. Furthermore, conformational differences between the G51D PD and MSA α-syn aggregates were observed, and these distinctions persisted upon propagation in TgM83 mice. Our results indicate that, despite the co-existence of Lewy body and GCI pathology in G51D PD cases, the α-syn strain specified by the G51D *SNCA* mutation more closely resembles a PD-associated rather than an MSA-associated strain.

## Materials and methods

### Tissue samples

Details of the brain samples used are provided in Table 1. Frozen tissue from the temporal cortex (2^nd^ temporal gyrus) of two PD cases with the G51D *SNCA* mutation (“G51D PD-1” and “G51D PD-2”) were obtained from the Queen’s Square Brain Bank. The clinical and neuropathological findings for the G51D PD-1 [“Case two (G51D)”] and G51D PD-2 [“Case three (G51D)”] samples have been previously described [32]. Frozen tissue from the substantia nigra of an MSA case (“MSA-1”) was provided by the Massachusetts Alzheimer’s Disease Research Center. Frozen temporal cortical samples showing prominent α-syn pathology from two cases of sporadic PD (“Spor. PD-1” and “Spor. PD-2”), one additional MSA case (“MSA-2”), and a non-neurodegenerative disease control case were obtained from the University Health Network Neurodegenerative disease Brain Collection (UHN-NBC). Informed consent was obtained for all cases at the point of tissue collection.

**Table 1:** Synucleinopathy patient samples

Sample	*SNCA* mutation	Sex	Age (years)	Brain region	Used for transmission experiments?
G51D PD-1	G51D	F	75	Temporal cortex (2nd gyrus)	Yes
G51D PD-2	G51D	M	52	Temporal cortex (2nd gyrus)	Yes
MSA-1	None	M	65	Substantia nigra	Yes
MSA-2	None	M	64	Substantia nigra	No
Sporadic PD-1	None	F	82	Temporal cortex	No
Sporadic PD-2	None	M	74	Temporal cortex	No
Control	None	F	26	Substantia nigra	No

### Mice

Two lines of mice were purchased from The Jackson Laboratory: homozygous TgM83 mice, which express A53T-mutant human α-syn under the control of the mouse prion protein promoter [22] on a mixed C57BL6/C3H background (stock number: 004479); and non-transgenic B6C3F1 mice (stock number: 100010). These two lines were intercrossed to generate the hemizygous TgM83^+/-^ mice used for inoculation experiments. TgM83^+/-^ mice were housed in groups of 4–5 animals per cage and were maintained on a 12 h light/12 h dark cycle. Mice had free and unlimited access to food and water. All studies utilized roughly equal numbers of male and female animals, though intercurrent illness required the removal of several mice from subsequent analysis (Additional file 1: Table S1).

### Tissue homogenization and sample preparation

Brain homogenates from frozen human tissue or TgM83^+/-^ mice [10% (w/v)] were generated by homogenization in calcium- and magnesium-free phosphate-buffered saline (PBS) using a Minilys homogenizer and CK14 soft tissue homogenizing tubes (Bertin). Homogenates were aliquoted and stored at -80 °C for later analysis. To analyze total protein levels, nine volumes of brain homogenate were combined with one volume of 10X detergent buffer [5% (v/v) Nonidet P-40, 5% (w/v) sodium deoxycholate, prepared in PBS] with added Pierce Universal Nuclease (ThermoFisher #88,701) and Halt Phosphatase Inhibitor (ThermoFisher #784,420) before incubation on ice for 20 min with occasional vortexing. Detergent-extracted brain extracts were clarified via centrifugation at 1,000 × *g* for 5 min at 4 °C prior to further use.

### Intracerebral inoculations

Intracerebral inoculation experiments were performed on ~ 5-week-old TgM83^+/-^ mice. Groups of 8–10 mice were anaesthetized using isoflurane gas and then inoculated non-stereotactically using a tuberculin syringe with an attached 27 gauge, 0.5-inch needle (BD Biosciences #305945) with 30 μL of sample [1% (w/v) crude human brain homogenate diluted in PBS containing 5% (w/v) BSA] to a depth of ~ 3 mm into the right cerebral hemisphere of the brain. This region roughly corresponds to the hippocampus and overlying cortex. Once inoculated, mice were monitored daily for general health and 2–3 times per week for signs of neurological disease. Mice were euthanized when they exhibited signs of end-stage disease – prominent hindlimb paralysis with reduced ambulation accompanied by weight loss and kyphosis [34]. For euthanasia, mice underwent transcardiac perfusion with 0.9% saline solution and brains were collected and divided parasagittally. The left hemisphere was frozen and stored for biochemical analysis at -80 °C while the right hemisphere was fixed in 10% neutral buffered formalin and stored at room temperature for later neuropathological examination.

### SDS-PAGE and immunoblotting

Samples were prepared in 1X Bolt LDS sample buffer prior to boiling. Gel electrophoresis was performed using 4–12% or 12% Bolt Bis–Tris Plus gels (Thermo Scientific) for 35–40 min at 165 V. Proteins were transferred to 0.45 μm polyvinylidene fluoride membranes immersed in transfer buffer [25 mM Tris, pH 8.3, 0.192 M glycine, 20% (v/v) methanol] for 1 h at 35 V. Proteins were crosslinked to the membrane via incubation in 0.4% (v/v) paraformaldehyde in PBS for 30 min at room temperature, with rocking [37]. Membranes were blocked for 1 h at room temperature in blocking buffer [5% (w/v) skim milk in 1X TBST (Tris-buffered saline containing 0.05% (v/v) Tween-20)] and then incubated overnight at 4 °C with primary antibodies diluted in blocking buffer. Primary antibodies used were anti-Serine 129-phosphorylated α-syn (PSyn) rabbit monoclonal EP1536Y (Abcam #ab51253; 1:10,000 dilution), anti-α-syn mouse monoclonal Syn-1 (BD Biosciences #610786; 1:10,000 dilution), and anti-actin 20–33 (Millipore Sigma #A5060; 1:10,000 dilution). Membranes were washed 3 times with TBST and then incubated, for 1 h at room temperature, with horseradish peroxidase-conjugated secondary antibodies (Bio-Rad #172–1019 or 172–1011) diluted 1:10,000 in blocking buffer. Following another 3 washes with TBST, immunoblots were developed using Western Lightning ECL Pro (PerkinElmer) or SuperSignal West Dura (ThermoFisher) and imaged using X-ray film or the LiCor Odyssey Fc system.

### Detergent insolubility assays and thermolysin digestion of α-syn aggregates

Detergent-extracted brain homogenates (200 µg) were diluted using 1X detergent buffer [0.5% (v/v) Nonidet P-40, 0.5% (w/v) sodium deoxycholate in PBS]. To generate detergent-soluble and detergent-insoluble fractions, samples were vortexed and then subjected to ultracentrifugation at 100,000 × *g* for 1 h at 4 ºC in a TLA-55 rotor (Beckman Coulter). Supernatants were removed and used as the detergent-soluble fraction. For the detergent-insoluble fraction, pellets were resuspended in 1X LDS loading buffer and then boiled for 10 min at 95 ºC prior to gel electrophoresis and immunoblotting, as described above. For thermolysin digestions, detergent-extracted brain homogenate was diluted into 1X detergent buffer containing 50 µg/mL thermolysin (Millipore Sigma #T7902). Samples were incubated at 37 °C with continuous shaking (600 RPM) for 1 h. Digestions were halted with the addition of EDTA to a final concentration of 5 mM, and samples were ultracentrifuged at 100,000 × *g* for 1 h at 4 °C. The supernatant was discarded and pellets were resuspended via boiling in 1X Bolt LDS loading buffer containing 2.5% (v/v) β-mercaptoethanol. Samples were then analyzed via SDS-PAGE followed by immunoblotting, as described above.

### Conformational stability assays of α-syn aggregates

α-Syn conformational stability assays were performed as previously described [34, 35]. Guanidine hydrochloride (GdnHCl) was added to detergent-extracted brain homogenate to yield final GdnHCl concentrations of 1, 2, 2.5, 3, 3.5, 4, 4.5, 5, 5.5 and 6 M in a volume of 40 µL. Samples were incubated at room temperature with shaking for 2 h (800 RPM) before being diluted to 0.4 M GdnHCl in detergent buffer. Following ultracentrifugation at 100,000 × *g* for 1 h at 4 ºC in a TLA-55 rotor, pellets were resuspended in 1X LDS loading buffer and boiled for 10 min. Samples were then analyzed via SDS-PAGE followed by immunoblotting, as described above.

### Neuropathological analysis

Formalin-fixed mouse hemibrains were dehydrated via immersion in a graded series of ethanol concentrations using an automated tissue processor prior to paraffin wax embedding. Brains were sagittally sectioned into 5 μm slices at the brain midline (~ 0.5 – 1 mm lateral) and mounted on glass slides. For immunohistochemistry, slides were heated to 60 °C to melt the paraffin, then deparaffinized and rehydrated using a graded xylene-to-ethanol series. Endogenous peroxidase activity was quenched by immersing the slides in 3% (v/v) H_2_O_2_ prepared in methanol for 25 min, followed by three 5-min rinses with PBS containing 0.05% (v/v) Tween-20 (PBST). Sections were blocked by incubating in 2.5% (v/v) normal horse serum from the ImmPRESS HRP detection system (Vector Laboratories #MP740150) for 1 h at room temperature. Immunohistochemical staining was performed with the rabbit monoclonal antibody EP1536Y (Abcam #ab51253; 1:320,000 dilution), which recognizes PSyn. Primary antibodies were diluted in antibody diluent (Dako #S080983-2) and staining was performed overnight at 4 °C. After three 5-min rinses with PBST, the slides were incubated for 30 min with anti-rabbit IgG from the ImmPRESS HRP detection system. Slides were then rinsed 3 times for 5 min with PBST and developed with ImmPACT 3,3’-diaminobenzidine (Vector Laboratories #SK4105) for 1 min and submerged in water to stop the reaction. After rinsing for 10 min under running tap water, the slides were counterstained with haematoxylin (Millipore Sigma #GHS132), submerged in water to stop the reaction, and then rinsed under running tap water again for 10 min. Slides were dehydrated with a graded ethanol-to-xylene series, then air-dried and mounted using Cytoseal 60 mounting solution (ThermoFisher #8310–4). Slides were either photographed using a Leica DM6000B microscope (63X objective) or were digitized using the TissueScope LE120 slide scanner and the TissueSnap preview station (Huron Digital Pathology).

For quantification of PSyn pathology, the number of PSyn-positive inclusions in a single sagittal brain section was manually counted in the parahippocampal region (including the corpus callosum) and the base of the brain in proximity to the diagonal band nucleus and preoptic area. PSyn deposition in the hypothalamus, midbrain, and brainstem was quantified by taking snapshots of the respective areas from scanned slides of single sagittal brain sections. Positively stained areas were isolated using ImageJ and the IHC Toolbox plugin (H-DAB model), images were converted to 8-bit black and white, the threshold was adjusted (range: 0–130), and then the percentage of the area covered was measured.

For double fluorescent labeling of PSyn and NeuN, slides were deparaffinized, rehydrated, and then subjected to heat-induced epitope retrieval for 15 min in citrate buffer pH 6. After blocking, slides were incubated with EP1536Y (1:10,000 dilution) and the anti-NeuN mouse monoclonal antibody 1B7 (Abcam #104224; 1:500 dilution) overnight at 4 °C. The following fluorescently labeled secondary antibodies (Thermo Fisher) were used: AlexaFluor488-conjugated goat anti-mouse (1:2,000 dilution) and AlexaFluor594-conjugated goat anti-rabbit (1:1,000 dilution). Slides were mounted using DAKO fluorescence mounting medium, sealed with clear nail polish, and then imaged using a Leica DM6000B microscope (63X objective).

### α-Syn seed amplification assays (SAAs)

To generate seeds for SAA reactions, 10% (w/v) brain homogenates (in PBS) were centrifuged at 10,000 × *g* for 10 min at 4 °C. The supernatant was collected, and the protein concentration in the PBS-soluble fraction was determined using the BCA assay. α-Syn SAA reactions were performed in black 384-well plates with a clear bottom (ThermoFisher Scientific #142761). Recombinant wild-type human α-syn (rPeptide #S-1001–2) was thawed from −80 °C storage, reconstituted in HPLC-grade water (MilliporeSigma) and filtered through a 100-kDa spin filter (Thermo Scientific). For the reaction mixture, each well contained 10 μL of the seed (5 μg of total protein from the PBS-soluble fraction, diluted in PBS), 10 μL of 50 μM Thioflavin T (ThT; final concentration: 10 µM), 10 μL of 0.5 mg/mL monomeric recombinant α-synuclein (final concentration: 0.1 mg/mL), and 10 µL of the appropriate reaction buffer (from 5X concentrated stock solutions). The PD-enhanced buffer contained 50 mM glycine pH 4, 50 mM NaClO_4_ whereas the MSA-enhanced buffer consisted of 40 mM phosphate buffer pH 8, 350 mM Na_3_Citrate. Plates were sealed and incubated at 37 °C in a BMG FLUOstar Omega plate reader with cycles of 1 min shaking (400 RPM double orbital) and 14 min rest for a period of 42 h. ThT fluorescence measurements (450 ± 10 nm excitation and 480 ± 10 nm emission, bottom read) were taken every 15 min. For human samples, 4–6 technical replicates were analyzed per case; for mouse samples, 3–4 technical replicates were analyzed per mouse brain sample. A replicate was defined as positive if there was a sustained increase in fluorescence over the baseline value and a fluorescence value of at least 4000 RFU was achieved within the 42 h.

To calculate maximum ThT fluorescence values, only samples that were positive and reached a fluorescence plateau within the experimental timeframe were included. For human samples, maximum ThT values for individual technical replicates were used. For mouse samples, maximum ThT values for individual technical replicates were averaged to generate a single value for each mouse. The kinetic curves for amplifications with the mouse samples were fit to a sigmoidal dose–response (variable slope) model in GraphPad Prism to obtain values for the Hill slope (k) and the time at which fluorescence is halfway between the baseline and plateau values (T_50_). Lag phases were then calculated using the equation T_50_ – [1/(2*k)] [1]. For samples in which a fluorescence plateau was not reached (i.e., those that started to aggregate towards the end of the experiment), the fit was constrained so that the “Top” fluorescence value could not exceed the fluorescence value at 42 h. Samples that did not aggregate were assigned a lag phase of 42 h. Lag phases for individual technical replicates were averaged to generate a single value for each mouse.

For SAAs using G51D-mutant α-syn, full-length, untagged human G51D-mutant α-syn was cloned into the pET-28a vector and expressed and purified from *E. coli* Rosetta 2(DE3) (Millipore Sigma #71400–3) using a modified osmotic shock and anion exchange protocol [29]. Following induction of protein expression using isopropyl β-D-1-thiogalactopyranoside for 3 h, cell pellets were resuspended in osmotic shock buffer (30 mM Tris–HCl pH 7.2, 40% sucrose, 2 mM EDTA). After incubation at room temperature for 10 min, the suspension was centrifuged at 9,000 × *g* for 20 min at 20 °C. The supernatant was discarded, and the pellet was resuspended in ice-cold dH_2_O, followed by the addition of saturated MgCl_2_ (40 μL per 100 mL of bacterial cell suspension) and allowed to incubate on ice for 3 min. The suspension was then centrifuged at 9,000 × *g* for 30 min at 4 °C, and the supernatant was collected, filtered through a 0.22 μm filter, and then dialyzed into 50 mM Tris–HCl pH 8.3 overnight at 4 °C. Recombinant α-syn was first purified using a HiPrep Q HP column (Cytiva Life Sciences) and eluted using a linear gradient of 0 to 500 mM NaCl in 50 mM Tris–HCl pH 8.3. Fractions containing sufficiently pure α-syn were pooled and re-dialyzed into 50 mM Tris–HCl pH 8.3 overnight at 4 °C. Fractions were further purified using a Mono Q column (Cytiva Life Sciences) and eluted using the same linear gradient. Fractions containing pure α-syn were dialyzed into dH_2_O and protein concentration was determined by measuring absorbance at 280 nm using a NanoDrop spectrophotometer (extinction coefficient = 5960). Recombinant α-syn was diluted to 0.5 mg/mL, aliquoted, flash frozen, and then stored at -80 °C. α-Syn SAAs were performed as described above using the MSA-enhanced buffer, except that reactions were performed in 96-well clear-bottom black microplates (Thermo Fisher #165305) containing 2 µg of PBS-soluble protein in a final volume of 100 µL.

### Statistical analysis

All statistical analysis was performed using GraphPad Prism (v.9.3) with a significance threshold of *P* = 0.05. Data comparisons were made using either one-way ANOVA with Tukey’s multiple comparisons test, two-way ANOVA with Tukey’s multiple comparisons test, the Kruskal–Wallis test followed by Dunn’s multiple comparisons test, or the Brown-Forsythe ANOVA followed by Dunnett’s T3 multiple comparisons test.

## Results

### G51D PD α-syn aggregates do not induce overt clinical disease in TgM83^+/-^ mice

**Fig. 1:**
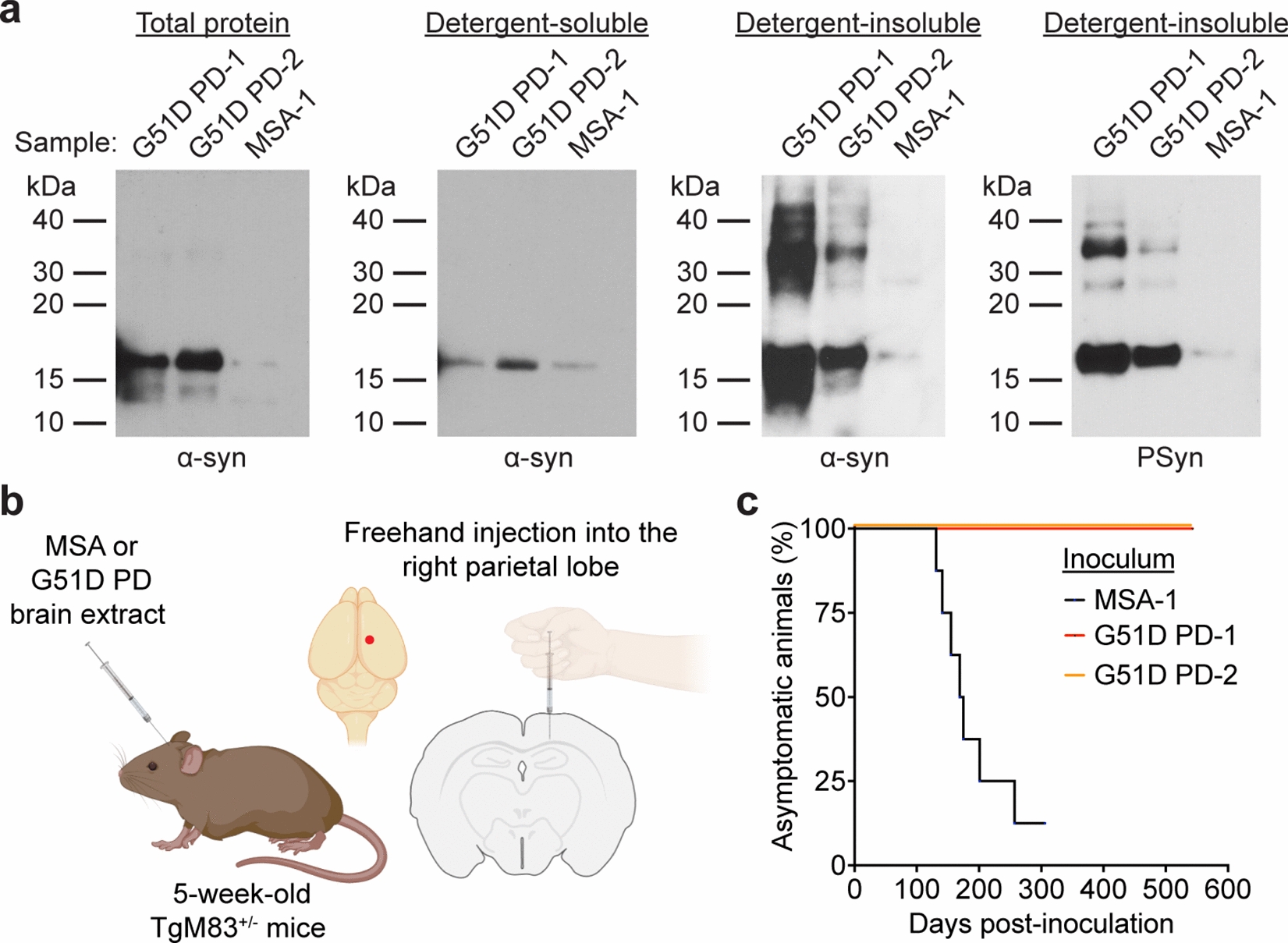
Inoculation of G51D PD and MSA samples into TgM83^+/-^ mice. **a** Immunoblots of total α-syn, detergent-soluble α-syn, detergent-insoluble α-syn, and detergent-insoluble PSyn species in brain homogenates from the G51D PD-1, G51D PD-2, and MSA-1 cases. α-Syn was detected using the antibody Syn-1 and PSyn was detected using the antibody EP1536Y. **b** Schematic of propagation studies in TgM83^+/-^ mice involving intracerebral inoculation of brain homogenates from G51D PD or MSA cases. The red dot indicates the approximate inoculation site. **c** Kaplan–Meier survival curves for TgM83^+/-^ mice inoculated with G51D PD-1 (red, n = 6), G51D PD-2 (orange, n = 5), or MSA-1 (black, n = 8) brain homogenate. There was a significant difference in the survival curves as determined by the Log-rank test (*P* = 0.00030)

Prion strains are known to produce distinct and heritable effects when injected into mice. We hypothesized that α-syn aggregates from MSA and G51D PD patients, due to overlapping neuropathological and clinical symptoms in their respective diseases, may exhibit similar biochemical characteristics and behavior upon transmission to mice. To test this, we conducted intracerebral inoculation experiments using brain tissue derived from two G51D PD patients and one with MSA (Table 1), hereafter referred to as G51D PD-1, G51D PD-2 and MSA-1, respectively. For the G51D PD patients, samples were obtained from the temporal cortex. Previous neuropathological characterization of these cases revealed prominent neuronal α-syn pathology and neuronal loss in this brain region, along with a lower extent of GCI-like pathology [32]. To control for the lower proportion of glial-associated pathology in these samples, the MSA-1 tissue was sourced from the substantia nigra, which also possesses lower levels of GCI pathology [47]. Levels of detergent-insoluble α-syn and PSyn, the latter of which is an indicator of pathological α-syn [21], were much higher in the two G51D PD cases compared to the MSA-1 case (Fig. 1a). Notably, levels of both detergent-insoluble PSyn and detergent-insoluble total α-syn were higher in the G51D PD-1 case than the G51D PD-2 case.

We performed transmission experiments in TgM83 mice, which over-express human α-syn containing the PD-causing A53T mutation. In their homozygous form, M83 mice develop spontaneous clinical illness beginning around 8 months of age [22]. Hemizygous M83 mice (TgM83^+/-^), on the other hand, do not develop disease for up to 20 months of age, providing a window in which accelerated disease onset may be observed following the cerebral introduction of α-syn seeds. For the transmission experiments, crude brain homogenates in PBS (without any further α-syn extraction) were intracerebrally inoculated into the brains of TgM83^+/-^ mice using a freehand inoculation method [34, 51, 80], which predominantly delivers α-syn seeds to the hippocampus as well as to overlying cortical regions (Fig. 1b). The appearance of signs of neurological illness, typified by weight loss, bradykinesia, and hindlimb paralysis, were then monitored longitudinally [34, 42, 46, 80]. A subset of inoculated mice died without exhibiting neurological illness or were euthanized due to intercurrent illness (Additional file 1: Table S1) and were therefore excluded from our analysis. In agreement with our previous findings [34], this predominantly occurred in male animals. None of the TgM83^+/-^ mice inoculated with the G51D PD samples, referred to hereafter as “G51D PD-1 mice” and “G51D PD-2 mice”, developed signs of neurological illness for up to 18 months post-inoculation (Fig. 1c). In contrast, 7 of 8 mice inoculated with MSA-1, referred to hereafter as “MSA mice”, presented with overt signs of clinical disease with a mean onset of 176 ± 16 days post-inoculation (DPI), consistent with previous MSA transmissions in the TgM83^+/-^ line [34, 51, 80]. These results suggest that the presence of GCI-like pathology in the samples from human G51D PD cases is insufficient to elicit clinical disease in TgM83^+/-^ mice.

### TgM83^+/-^ mice inoculated with G51D PD develop a subclinical synucleinopathy

**Fig. 2:**
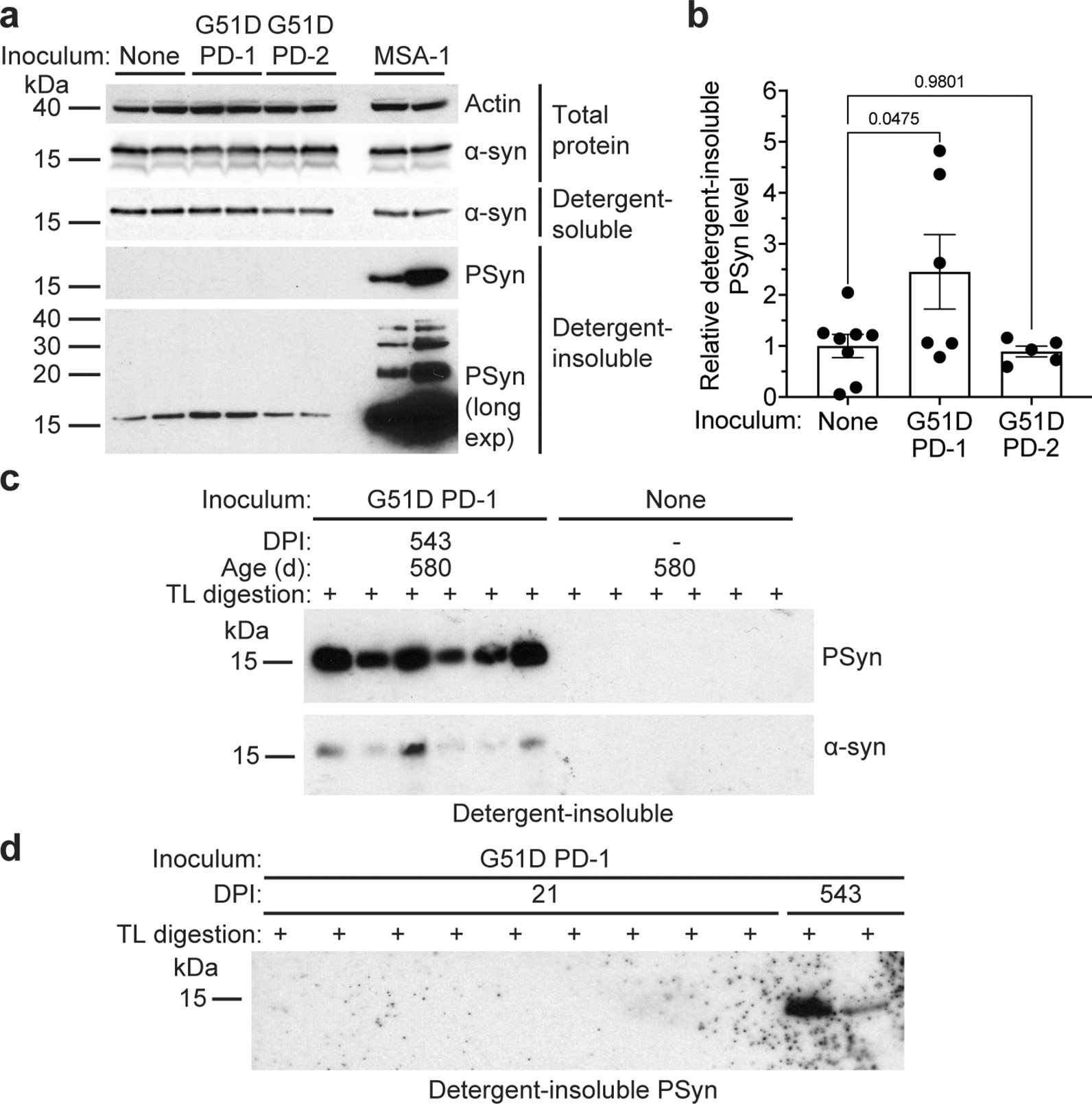
Biochemical detection of α-syn aggregates in the brains of inoculated TgM83^+/-^ mice. **a** Immunoblots of total α-syn, detergent-soluble α-syn, and detergent-insoluble PSyn species in brain homogenates from G51D PD-1 and G51D PD-2 mice at 540–543 DPI, clinically ill MSA mice at 155–257 DPI, as well as uninoculated TgM83^+/-^ mice at 580 days of age. The blot of total α-syn was reprobed with an antibody against actin. **b** Quantification of detergent-insoluble PSyn levels (mean ± s.e.m.) in the brains of G51D PD-1 (n = 6) and G51D PD-2 (n = 5) mice at 540–543 DPI compared to age-matched uninoculated TgM83^+/-^ mice (n = 8). *P* values were calculated using one-way ANOVA followed by Dunnett’s multiple comparisons test. **c** Immunoblot of detergent-insoluble, thermolysin (TL)-resistant PSyn and total α-syn levels in brain homogenates from G51D PD-1 mice at 543 DPI or age-matched uninoculated TgM83^+/-^ mice (n = 6 each). **d** Immunoblot of detergent-insoluble, TL-resistant PSyn levels in brain homogenates from G51D PD-1 mice at either 21 (n = 9) or 543 (n = 2) DPI. In panels a, c, and d, PSyn was detected using the antibody EP1536Y and α-syn was detected using the antibody Syn-1

While there were no apparent differences in the levels of total and detergent-soluble α-syn between the experimental groups, significantly higher levels of detergent-insoluble PSyn were observed in the brains of asymptomatic G51D PD-1 mice at 18 months post-inoculation compared to a cohort of age-matched uninoculated TgM83^+/-^ mice (Fig. 2a, b) [34]. Levels of insoluble PSyn in G51D PD-2 mice were not increased compared to the uninoculated mice, possibly due to the lower amount of insoluble PSyn in the G51D PD-2 inoculum (Fig. 1a). Levels of detergent-insoluble PSyn were substantially increased in the brains of clinically ill MSA mice compared to the uninoculated and G51D PD-1 mice (Fig. 2a). We estimate that PSyn levels were approximately 40 times higher in the MSA mice compared to the G51D PD-1 mice (Additional file 1: Figure S1). We have previously shown that digestion of brain homogenates with the protease thermolysin (TL) can be used to assess the presence of α-syn aggregates in TgM83^+/-^ mice [34]. Despite being asymptomatic, all 6 of the G51D PD-1 mice at 18 months post-inoculation displayed TL-resistant, insoluble α-syn aggregates in their brains, whereas none of the 6 age-matched uninoculated mice exhibited TL-resistant α-syn (Fig. 2c). To confirm that the signal we observed in mice at 18 months post-inoculation did not represent persistence of the G51D PD-1 inoculum, we inoculated a separate cohort of TgM83^+/-^ mice with the G51D PD-1 sample and analyzed their brains at 3 weeks following injection. No TL-resistant α-syn was observed in the brains of these mice, arguing that the α-syn aggregates in the brains of G51D PD-1 mice at 18 months post-inoculation derive from the inoculum-induced conversion of host-encoded α-syn (Fig. 2d).

### MSA- and G51D PD-inoculated TgM83^+/-^ mice develop distinct synucleinopathies

**Fig. 3:**
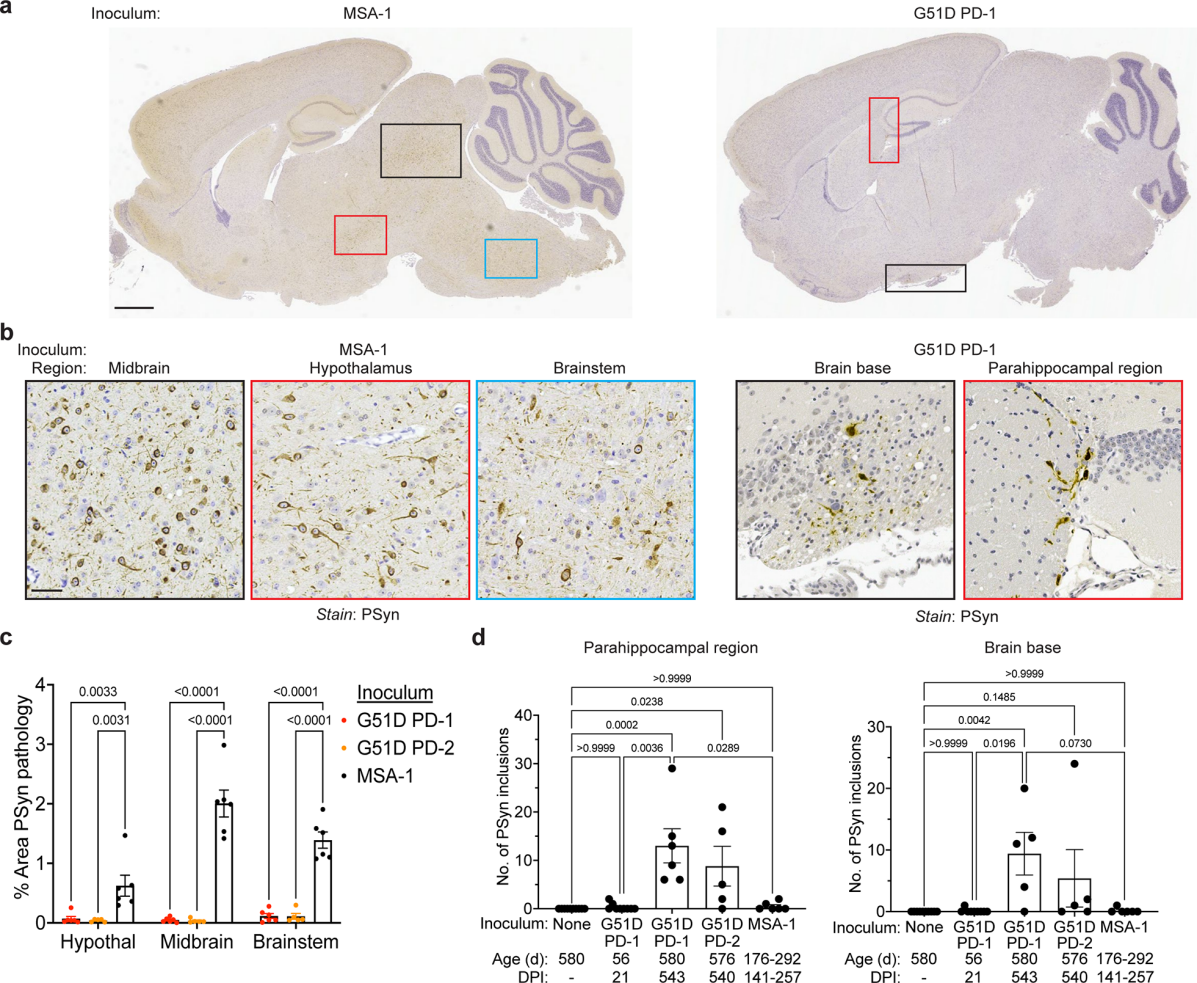
G51D PD and MSA produce distinct patterns of PSyn deposition in TgM83^+/-^ mice. **a** Images of PSyn-stained brain sections (EP1536Y antibody) from a clinically ill MSA mouse at 257 DPI and an asymptomatic G51D PD-1 mouse at 543 DPI. Colored boxes indicate areas of the brain shown in higher magnification in panel b. Scale bar = 1 mm (applies to both images). **b** Images of the midbrain (black border), hypothalamus (red border), and brainstem (blue border) from a clinically ill MSA mouse at 257 DPI as well as the brain base (black border) and parahippocampal region (red border) from an asymptomatic G51D PD-1 mouse at 543 DPI. All sections were stained with the EP1536Y PSyn antibody. Scale bar = 50 µm (applies to all images). **c** Quantification of PSyn deposition in the hypothalamus, midbrain, and brainstem of G51D PD-1 mice at 543 DPI (red, n = 6), G51D PD-2 mice at 540 DPI (orange, n = 5), or clinically ill MSA mice at 141–257 DPI (black, n = 6). Data is mean ± s.e.m. *P* values were calculated using a two-way ANOVA with Tukey’s multiple comparisons test. **d** Quantification of the number of PSyn deposits in the parahippocampal region (left) and brain base (right) of aged uninoculated TgM83^+/-^ mice (n = 10), G51D PD-1 mice at either 21 DPI (n = 9) or 543 DPI (n = 6 for parahippocampal, n = 5 for brain base), G51D PD-2 mice at 540 DPI (n = 5), and clinically ill MSA mice at 141–257 DPI (n = 6). Data is mean ± s.e.m. *P* values were calculated using a Kruskal–Wallis test followed by Dunn’s multiple comparisons test

Immunohistochemical analysis of brains from clinically ill MSA mice using an antibody against PSyn that does not cross-react with phosphorylated neurofilaments [57] revealed extensive α-syn pathology (Fig. 3a, b, Additional file 1: Figure S2), consistent with the robust induction of clinical disease in this cohort. In MSA mice, the majority of the PSyn deposition was observed in regions of the hindbrain, most notably the midbrain, hypothalamus, and brainstem, similar to what has been described previously [13, 80, 82]. In MSA mice, significantly higher amounts of PSyn pathology were present in the hypothalamus, midbrain, and brainstem compared to G51D PD-1 and G51D PD-2 mice (Fig. 3c). Asymptomatic G51D PD-1 mice at 18 months post-injection exhibited a more restricted pattern of cerebral PSyn staining, with most of the pathology observed in the parahippocampal region (including the corpus callosum) and the base of the brain in proximity to the diagonal band nucleus and preoptic area (Fig. 3a, b, Additional file 1: Figure S2). Indeed, significantly higher numbers of PSyn deposits in these regions were observed in mice at 18 months than at 3 weeks post-inoculation with G51D PD-1, indicating that the PSyn deposition was progressive (Fig. 3d). Furthermore, PSyn deposits were found in the parahippocampal region of two G51D PD-1 mice euthanized at 235 DPI (Additional file 1: Figure S3). No PSyn staining was observed in any of the aged uninoculated TgM83^+/-^ mice in the parahippocampal and brain base regions (Fig. 3d). Moreover, despite an overall higher burden of PSyn pathology, significantly fewer PSyn deposits were present in the parahippocampal region of MSA mice compared to G51D PD-1 mice. PSyn pathology was also found in the brains of G51D PD-2 mice at 18 months post-inoculation, albeit to a lesser and more variable extent (Fig. 3d, Additional file 1: Figure S2). Occasional PSyn deposits in the brainstem, midbrain, and thalamus were observed in a subset of the G51D PD-1 mice (Additional file 1: Figure S4).

**Fig. 4:**
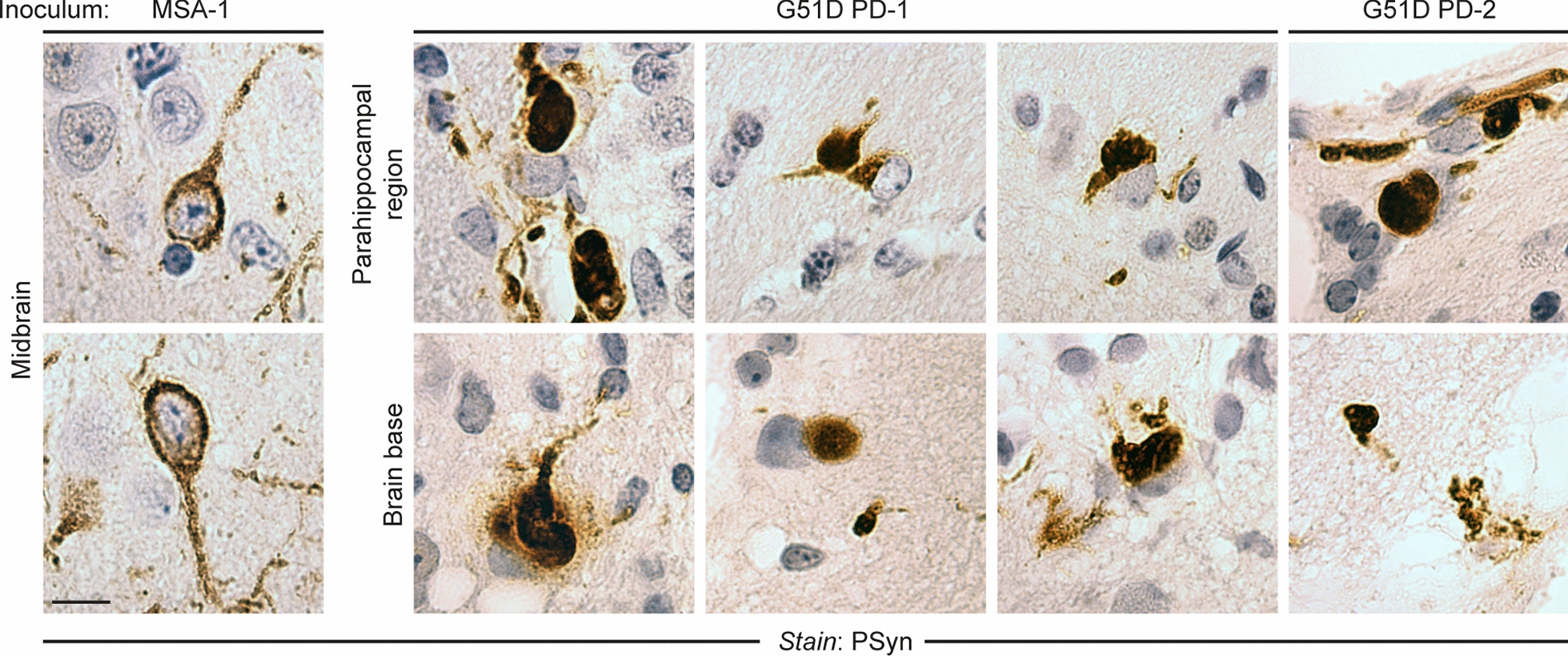
G51D PD and MSA produce distinct PSyn aggregate morphologies in TgM83^+/-^ mice. Images of PSyn inclusions in the indicated brain regions of a symptomatic MSA mouse (257 DPI) or asymptomatic G51D PD-1 and G51PD-2 mice at 540–543 DPI. Mice injected with MSA display “ring-like” PSyn inclusions whereas mice inoculated with G51D PD exhibit rounded, dense PSyn inclusions. The images from G51D PD-1 mice are derived from 5 distinct animals and the images from G51D PD-2 mice are derived from 2 distinct animals. All sections were stained with the EP1536Y PSyn antibody. Scale bar = 10 µm (applies to all images)

In MSA mice, the PSyn pathology was neuronal and exhibited a “ring-like” morphology that filled the cytoplasm and extended into the cellular processes, similar to what we have previously described [34] (Fig. 4, Additional file 1: Figure S5). The morphology of the induced PSyn deposits in the brains of G51D PD-1 and G51D PD-2 mice was distinct from those present in the MSA mice. Many of the PSyn deposits in G51D PD-1 mice were much denser and more rounded (Fig. 4). Similar types of deposits were observed in the G51D PD-2 mice. Collectively, these results indicate that MSA and G51D PD brain extracts induce distinct synucleinopathies upon inoculation of TgM83^+/-^ mice, characterized by differences in the patterns of PSyn deposition and morphologies of the induced α-syn aggregates.

### G51D PD- and MSA-associated α-syn aggregates exhibit distinct seeding behaviors

**Fig. 5:**
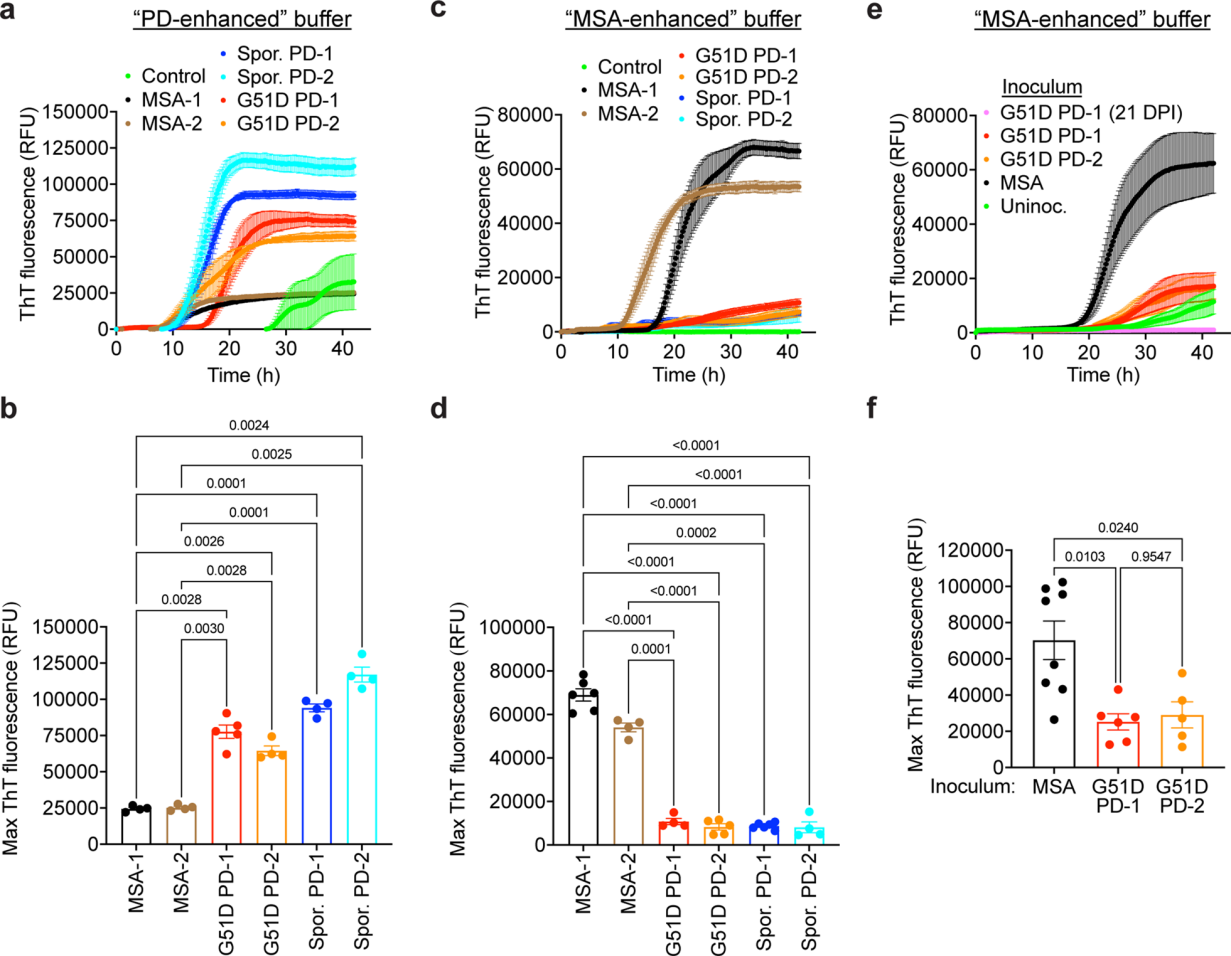
G51D PD-derived α-syn aggregates possess seeding properties more reminiscent of sporadic PD than MSA. **a** ThT fluorescence curves for α-syn SAA experiments using a PD-enhanced buffer on α-syn aggregates from G51D PD, MSA, sporadic PD, and control human brain homogenates. Monomeric wild-type human α-syn was seeded with either G51D PD-1 (red), G51D PD-2 (orange), MSA-1 (black), MSA-2 (brown), sporadic PD-1 (dark blue), sporadic PD-2 (light blue), or control (green) brain extract. Each data point represents the mean ThT fluorescence ± s.e.m. from 4 to 6 technical replicates. **b** Maximum ThT fluorescence values obtained during α-syn SAA with the PD-enhanced buffer using brain homogenates from the indicated human samples as seeds. Data is mean ± s.e.m. from 4 to 6 technical replicates. **c** ThT fluorescence curves for α-syn SAA experiments using an MSA-enhanced buffer on α-syn aggregates from G51D PD, MSA, sporadic PD, and human control brain homogenates. Each data point represents the mean ThT fluorescence ± s.e.m. from 4 to 6 technical replicates. **d** Maximum ThT fluorescence values obtained during α-syn SAA with the MSA-enhanced buffer using brain homogenates from the indicated human samples as seeds. Data is mean ± s.e.m. from 4 to 6 technical replicates. **e** ThT fluorescence curves for α-syn SAA experiments using an MSA-enhanced buffer on α-syn aggregates in brain homogenates from aged uninoculated TgM83^+/-^ mice (green, n = 10), G51D PD-1 mice at either 21 (pink, n = 9) or 543 (red, n = 6) DPI, G51D PD-2 mice at 540 DPI (orange, n = 5), or clinically ill MSA mice (black, n = 8). Data is mean ± s.e.m. from 3 to 4 technical replicates per mouse. **f** Maximum ThT fluorescence values obtained during α-syn SAA with the MSA-enhanced buffer using brain homogenates from TgM83^+/-^ mice injected with the indicated human samples as seeds. Data is mean ± s.e.m. from 3 to 4 technical replicates per mouse. For panels b, d, and f, *P* values were calculated using a Brown-Forsythe ANOVA test followed by Dunnett’s T3 multiple comparisons test

Building on our finding that G51D PD-associated α-syn aggregates do not behave like MSA α-syn aggregates in TgM83^+/-^ transmission experiments, we hypothesized that G51D PD α-syn would exhibit seeding properties more reminiscent of sporadic PD in an α-syn seed amplification assay (SAA) similar to the real-time quaking-induced conversion (RT-QuIC) assay used to amplify prion seeds [56]. The α-syn SAA is based on the ability of pre-existing α-syn aggregates to template the conversion of monomeric recombinant α-syn into fibrillar, β-sheet-rich structures capable of binding the dye Thioflavin T (ThT). By monitoring ThT fluorescence levels, α-syn seeding activity in biological samples can be assessed in real time [8, 15, 24, 54, 62]. A caveat of this approach is that not all types of α-syn aggregates react well with ThT [12]. We have recently optimized the α-syn SAA for the discrimination of MSA- and PD-associated α-syn aggregates based on the maximum ThT signal obtained following amplification using different buffers [44]. Using a “PD-enhanced” buffer that generates a higher signal with PD-derived α-syn, seeding with the two G51D PD cases produced maximum ThT fluorescence levels that were similar to those obtained when seeding with two sporadic PD samples (Fig. 5a, b). Conversely, seeding with two MSA cases generated significantly lower maximum ThT fluorescence values when using this specific buffer. In reactions seeded with a control brain sample, an increase in ThT fluorescence was eventually observed, likely because the low pH of the PD-enhanced buffer promotes spontaneous aggregation of α-syn. We next tested the amplification properties of the samples using an “MSA-enhanced” buffer that generates higher ThT fluorescence values with MSA-derived α-syn. Once again, seeding with the two G51D PD cases produced maximum ThT fluorescence values in the α-syn SAA that were similar to those obtained when seeding with two cases of sporadic PD, whereas seeding with two MSA cases generated significantly higher ThT fluorescence in the assay (Fig. 5c, d). No spontaneous α-syn aggregation was observed in reactions seeded with a control brain sample when using the MSA-enhanced buffer, which utilizes a more physiological pH. Attempts to perform α-syn SAAs using recombinant human α-syn containing the G51D mutation as the substrate were unsuccessful (Additional file 1: Fig. S6), perhaps because the G51D mutation attenuates the aggregation of α-syn in vitro [17]. Thus, G51D PD cases behave more similarly to sporadic PD than MSA in an α-syn SAA paradigm.

Since the amplification properties of the human G51D PD and MSA cases in the α-syn SAA were distinct, we wondered whether such differences were conserved upon propagation of the corresponding α-syn aggregates in TgM83^+/-^ mice. Seeding with brain extracts from clinically ill MSA mice produced significantly higher ThT fluorescence in the α-syn SAA using the MSA enhanced buffer compared to seeding with extracts from asymptomatic G51D PD-1 and PD-2 mice at 18 months post-injection (Fig. 5e, f). Brain extracts from G51D PD-1 mice at 3 weeks post-inoculation were negative in the α-syn SAA, indicating that the positive signals obtained with samples from aged G51D PD-1 mice did not manifest due to persistence of the inoculated human-derived α-syn aggregates in the mice (Fig. 5e). Moreover, lag phases in the α-syn SAA were significantly shorter when seeding with samples from G51D PD-1 mice at 18 months post-injection compared to samples from aged uninoculated TgM83^+/-^ mice, suggesting that the seeding activity observed in the G51D PD-1 mice is not driven by low-level spontaneous formation of α-syn aggregates in the brains of these mice at advanced ages (Fig. 5e, Additional file 1: Fig. S7a). Indeed, in the α-syn SAA, seeding activity in at least 50% of individual technical replicates was detected in only 20% of brain extracts (2 of 10) from aged uninoculated TgM83^+/-^ mice (Additional file 1: Fig. S7b). In contrast, for the G51D PD-1 and PD-2 mice, α-syn seeding activity in at least half of the individual replicates was observed for 100% (6 of 6) and 80% (4 of 5) of the brain extracts, respectively. For the MSA mice, α-syn seeding was observed with every technical replicate from all mice (8 of 8) tested. Collectively, these results indicate that mice injected with G51D PD exhibit levels of α-syn seeding above background, and that the distinct amplification characteristics of MSA- and G51D PD-derived α-syn aggregates in the α-syn SAA are maintained upon propagation in TgM83^+/-^ mice.

### G51D PD- and MSA-associated α-syn aggregates are conformationally distinct

**Fig. 6:**
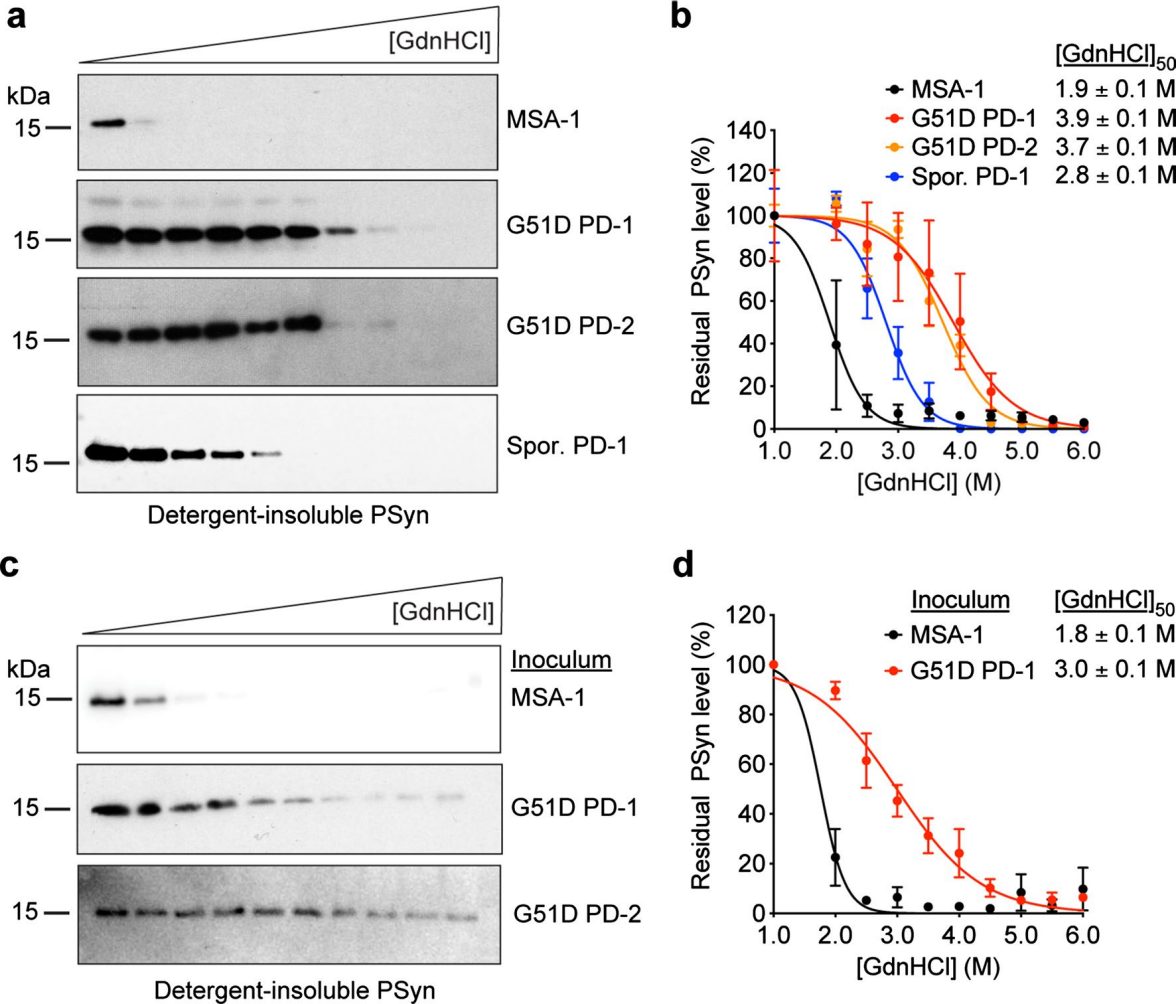
G51D PD-associated α-syn aggregates are more stable than MSA-associated α-syn aggregates. **a** Representative immunoblots from conformational stability assay analysis of α-syn aggregates in brain homogenates from human G51D PD, sporadic PD, and MSA samples. **b** Denaturation curves for residual PSyn levels (mean ± s.e.m. of 3–4 technical replicates per sample) following treatment with the indicated concentrations of GdnHCl. The calculated [GdnHCl]_50_ values for each sample (± standard error) are shown. **c** Representative immunoblots from conformational stability assay analysis of α-syn aggregates in brain homogenates from asymptomatic G51D PD-1 mice and G51D PD-2 mice at 540–543 DPI, or from a clinically ill MSA mouse. **d** Denaturation curves for residual PSyn levels (mean ± s.e.m. from n = 3 mice per inoculum) following treatment with the indicated concentrations of GdnHCl. The calculated [GdnHCl]_50_ values for α-syn aggregates within each experimental group (± standard error) are shown. In panels a and c, detergent-insoluble PSyn was detected using the antibody EP1536Y

Given the stark differences observed in the transmission and seeding properties of α-syn aggregates from G51D PD and MSA patient tissues, we assessed the conformational properties of the α-syn aggregates present in the human brain samples as well as the induced aggregates from the brains of inoculated TgM83^+/-^ mice. The conformational stability assay, which measures the solubilization of protein aggregates following treatment with increasing concentrations of guanidine hydrochloride (GdnHCl), can be used to distinguish α-syn strains [34, 35]. α-Syn aggregates in brain extracts from the human G51D PD-1 and G51D PD-2 cases as well as a case of sporadic PD were more stable than α-syn aggregates from the MSA-1 case (Fig. 6a, b). The calculated [GdnHCl]_50_ value – the concentration of GdnHCl at which 50% of aggregates have been solubilized – for the human MSA sample was similar to what we previously found for α-syn aggregates from MSA cases, whereas the values for the G51D PD and sporadic PD samples were more similar to those obtained for α-syn aggregates from cases of PD, DLB, and Alzheimer’s disease with concomitant Lewy body pathology [34, 44]. Mimicking the corresponding human cases, α-syn aggregates from TgM83^+/-^ mice inoculated with MSA were significantly less stable than α-syn aggregates from mice inoculated with G51D PD (Fig. 6c, d). Interestingly, while the stabilities of patient-derived and mouse-passaged MSA α-syn aggregates were similar, the stability of α-syn aggregates from the G51D PD-1 case appeared to decrease slightly upon propagation in TgM83^+/-^ mice. The reduced stability of α-syn aggregates in G51D PD-1 mice, which exhibited a [GdnHCl]_50_ value similar to α-syn aggregates in brain extract from a human sporadic PD case, potentially suggests that the G51D mutation helps to further stabilize PD-associated α-syn aggregates. Taken together, these results indicate that α-syn aggregates derived from MSA and G51D PD brain tissues are conformationally distinct and that these differences persist upon propagation in TgM83^+/-^ mice.

## Discussion

Given the presence of both PD- and MSA-like pathological hallmarks in the brains of individuals with the G51D *SNCA* mutation, we were interested in determining whether G51D PD-associated α-syn aggregates more closely resemble those from MSA or sporadic PD. Our results clearly demonstrate that G51D PD-associated α-syn aggregates exhibit conformational, seeding, and transmission properties more reminiscent of sporadic PD than MSA. In particular, like α-syn aggregates from PD patients, G51D PD α-syn aggregates produce ThT signals distinct from MSA α-syn aggregates in an α-syn SAA, are more resistant to denaturation with GdnHCl than MSA-associated α-syn aggregates, and fail to produce overt neurological illness upon inoculation into TgM83^+/-^ mice [34, 62, 80]. Although G51D PD-inoculated mice remained healthy for 18 months post-inoculation, a unique subclinical synucleinopathy was present in the brains of these animals, characterized by a more restricted distribution of α-syn pathology with preferential deposition in the parahippocampal region as well as the presence of denser and more rounded aggregates. Although we did not perform transmissions with sporadic PD samples in our study, the pathological phenotype in G51D PD-inoculated mice appears to be identical to the pattern of α-syn pathology recently described in asymptomatic TgM83^+/-^ mice at 18–19 months post-inoculation with sporadic PD brain homogenate [71], further supporting our contention that G51D PD α-syn aggregates are similar to those from cases of sporadic PD.

The presence of a localized, subclinical synucleinopathy in mice inoculated with G51D PD is consistent with the notion that the PD-associated α-syn strain is more slowly progressive than the MSA-associated α-syn strain. In G51D PD-1 mice, the induced α-syn pathology was predominantly found in brain areas adjacent to cerebral drainage regions and the ventricular system where the inoculum may pool post-injection. We hypothesize that α-syn seeds initially gain access to the ventricles following freehand inoculation into the right cerebral hemisphere, leading to widespread distribution throughout the brain via cerebrospinal fluid circulation pathways. We found that α-syn aggregates from the human G51D PD cases were much more stable than those from MSA, which may reduce their ability to fragment and thus restrict the generation of smaller seeds capable of spreading more widely into the brain parenchyma. Indeed, it has been found for both prion and α-syn aggregates that less stable aggregates propagate more rapidly than aggregates with a higher conformational stability [11, 34, 38]. Alternatively, the denser α-syn inclusions observed in G51D PD-1 mice could indicate that these aggregates are larger in size and therefore less able to transit from cell-to-cell within the brain. In contrast, while α-syn seeds from MSA cases likely infiltrate the brain from similar sites following inoculation, their lower stabilities may permit increased fragmentation and thus greater penetration into the brain, leading to higher levels of induced α-syn deposition deeper within the parenchyma. One study found that the extent of PSyn deposition in MSA-inoculated TgM83^+/-^ mice collected at 90 DPI was highly variable, with many mice exhibiting no midbrain or brainstem pathology at this timepoint [83]. This suggests that α-syn pathology develops and spreads rapidly as the disease course nears the clinical endpoint, potentially due to a threshold effect. While relative stability and propagation rate are clearly important, selective neuronal vulnerability may also be partially responsible for the distinct brain region tropisms of α-syn aggregates in mice inoculated with G51D PD or MSA brain extract. To this end, we have recently shown that two α-syn strains generated from recombinant α-syn target distinct brain regions and cell types [34].

It has been found that α-syn aggregates that originate in oligodendrocytes or have been propagated within the oligodendrocyte cellular milieu are more potent seeders of α-syn pathology than neuronally-derived α-syn strains [49]. However, we found that G51D PD temporal cortex extracts, derived from cases reported to contain both GCI-like oligodendroglial and LB-like neuronal α-syn aggregates in this brain region [32], produced neither clinical illness nor a widespread synucleinopathy upon propagation in TgM83^+/-^ mice. There are several potential explanations for this apparent contradiction. First, it is conceivable that the G51D PD brain samples we used did not contain sufficient oligodendroglial α-syn aggregates to elicit a response upon inoculation into TgM83^+/-^ mice. However, this is unlikely since even brain regions without robust GCI pathology from MSA patients transmit disease to TgM83^+/-^ mice within 200 days [83, 84]. Second, it is important to note that our study compared the behavior of MSA-associated GCIs (which are composed of aggregates containing wild-type human α-syn) and the GCI-like pathology seen in G51D PD patients (which likely consists of aggregates containing G51D-mutant α-syn). Despite these aggregates having been forged in the same glial milieu, the presence of the G51D mutation may have an attenuating effect on α-syn conversion kinetics, as seen in in vitro fibrillization studies [17, 59], or may impart a transmission barrier that prevents manifestation of the MSA-induced phenotype in TgM83^+/-^ mice that express A53T-mutant human α-syn. Third, the presence of abundant LB-like α-syn pathology in the G51D PD extracts may have interfered with the propagation of the GCI-like pathology, a phenomenon that has been observed with prion strains [4, 14, 63]. Finally, it is also possible that the GCI-like pathology observed in G51D PD may not truly be MSA-like at all and could simply represent the occurrence in oligodendrocytes of α-syn aggregates with similar conformational properties to PD-associated α-syn. It is known that sporadic PD cases do exhibit α-syn-positive oligodendrocytic inclusions within the midbrain [78], brainstem [61], and in the pallidothalamic tract, where the morphology of oligodendroglial α-syn inclusions is more voluminous [53]; however, white matter involvement in PD is believed to be limited [5].

While propagation of α-syn aggregates in TgM83^+/-^ mice was minimal following inoculation with G51D PD brain extract, others have observed more potent seeding effects following inoculation with α-syn PFFs containing the G51D mutation. G51D α-syn PFFs successfully induced α-syn pathology in non-transgenic mice [27] and rats [25], and decreased the time to onset of a clinical phenotype in homozygous TgM83 mice [58]. This suggests that the in vitro polymerization of recombinant G51D-mutant human α-syn into fibrils does not accurately mimic the aggregate structures formed in a human brain. Indeed, the structure of fibrils derived from recombinant G51D-mutant α-syn has recently been determined by cryogenic electron microscopy and is distinct from structures of wild-type recombinant human α-syn aggregates as well as α-syn aggregates isolated from the brains of individuals with sporadic PD [69, 86]. Although the effects of detergent treatment and other extraction techniques on the structural integrity and propagation properties of brain-derived α-syn strains remain to be fully deciphered, the structural differences between brain-derived and in vitro-polymerized α-syn aggregates imply that caution must be exercised when attempting to translate findings obtained using the latter to human diseases.

## Conclusions

In this study, we have demonstrated that the conformational and propagation properties of α-syn aggregates from PD patients with the G51D α-syn mutation are more reminiscent of α-syn aggregates from patients with sporadic PD than from patients with MSA. Thus, the G51D mutation specifies the formation of a slowly propagating α-syn strain.

## Supplementary Information

**Additional file 1**.

## Data Availability

The data used and/or analyzed during the current study are available from the corresponding author upon reasonable request.

## Abbreviations

DLB: Dementia with Lewy bodies
DPI: Days post-inoculation
GCI: Glial cytoplasmic inclusion
GdnHCl: Guanidine hydrochloride
LB: Lewy body
MSA: Multiple system atrophy
PBS: Phosphate-buffered saline
PD: Parkinson’s disease
PFFs: Pre-formed fibrils
PSyn: Serine 129-phosphorylated α-synuclein
SAA: Seed amplification assay
α-syn: α-Synuclein
TBST: Tris-buffered saline with Tween-20
ThT: Thioflavin T
TL: Thermolysin
