# Supplementary material for: The G51D SNCA mutation generates a slowly progressive α-synuclein strain in early-onset Parkinson’s disease
Source: Acta Neuropathol Commun. 2023 May 3;11:72. doi: 10.1186/s40478-023-01570-5 (PMC10155462; doi:10.1186/s40478-023-01570-5)
Supplement: Supplementary file 1 — Additional file 1. [file 40478_2023_1570_MOESM1_ESM.pdf]

Table S1. List of inoculated TgM83$^{+/-}$ mice with intercurrent illness removed from the study

Inoculum	ID	Sex	DPI	Notes
MSA-1	723	M	131	Died during cage change (no apparent neurological signs)
	724	M	148	Euthanized due to bladder stones
G51D PD-1	2106	M	235	Euthanized for fighting injury (no apparent neurological signs)
	2107	M	176	Found dead in cage (no apparent neurological signs)
	2108	M	224	Found dead in cage (no apparent neurological signs)
	2110	M	235	Euthanized for fighting injury (no apparent neurological signs)
G51D PD-2	1968	F	9	Found dead in cage (no apparent neurological signs)
	1974	M	309	Found dead in cage (no apparent neurological signs)
	1973	M	378	Euthanized for fighting injury (no apparent neurological signs)
	1975	M	169	Euthanized due to bladder stones
	1976	M	317	Euthanized for fighting injury (no apparent neurological signs)

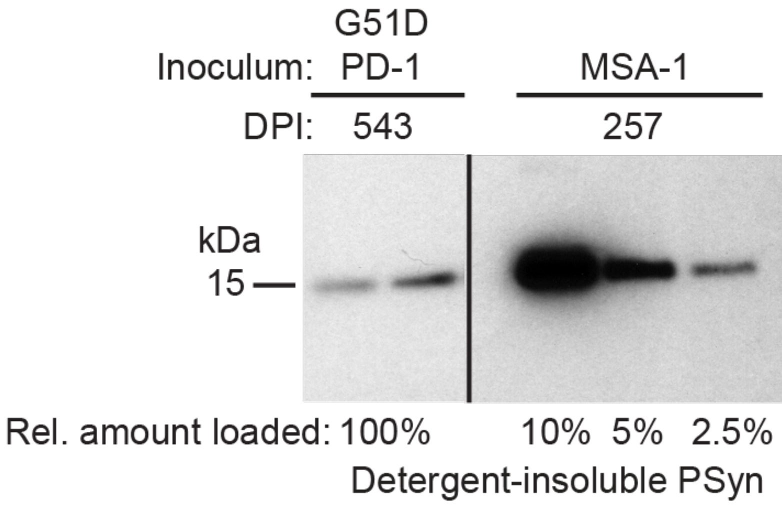

Fig. S1 Comparison of PSyn levels in the brains of mice inoculated with G51D PD or MSA. Immunoblot of detergent-insoluble PSyn levels in brain homogenates from TgM83$^{+/-}$ mice at the indicated DPI with either the G51D PD-1 or MSA-1 samples. The relative amount of protein loaded is given below the image. All samples were run on the same gel; the vertical line indicates removal of irrelevant lanes from the blot. PSyn was detected using the antibody EP1536Y

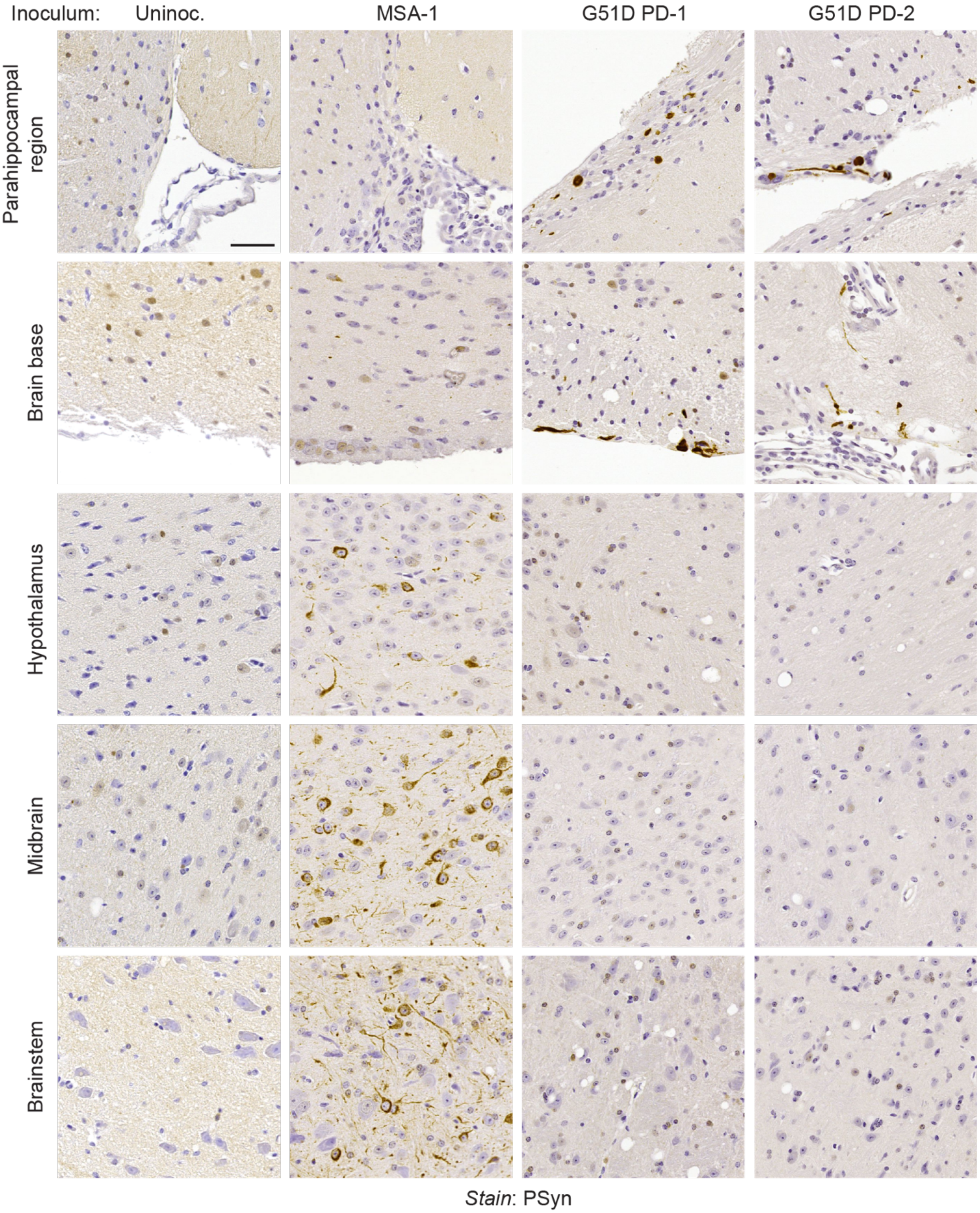

Fig. S2 Regional differences in PSyn pathology between TgM83$^{+/-}$ mice injected with MSA or G51D PD. Images of PSyn-stained sections (EP1536Y antibody) from the parahippocampal region, brain base, hypothalamus, midbrain, and brainstem of uninoculated TgM83$^{+/-}$ mice at 580 days of age, as well as MSA mice at 175 DPI, G51D PD-1 mice at 543 DPI, or G51D PD-2 mice at 540 DPI. Scale bar = 50 µm (applies to all images)

Inoculum: G51D PD-1
Parahippocampal region

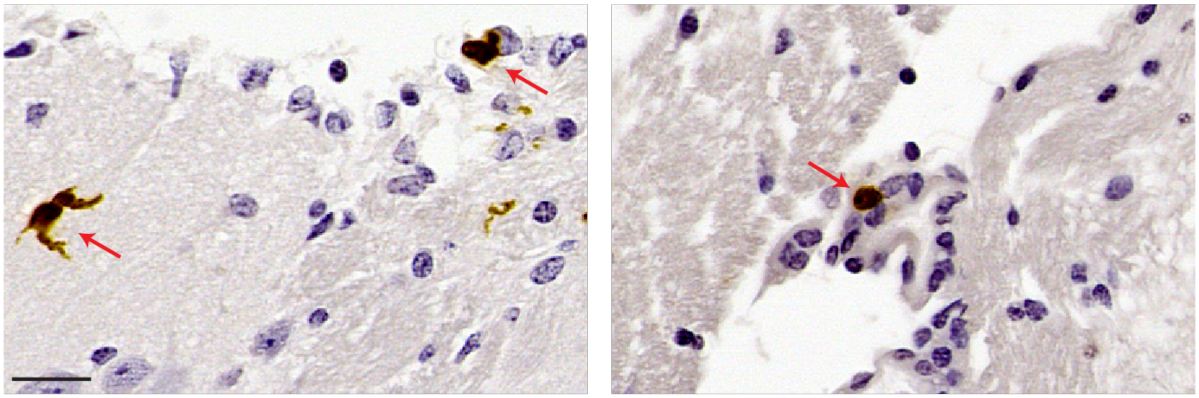

Stain: PSyn

Fig. S3 PSyn pathology in TgM83$^{+/-}$ mice at 235 DPI with G51D PD. Images of PSyn-stained brain sections (EP1536Y antibody) from the parahippocampal region of TgM83$^{+/-}$ mice at 235 DPI with the G51D PD-1 sample. The mice were euthanized due to intercurrent illness. The red arrows indicate PSyn deposits. Scale bar = 20 μm (applies to both images)

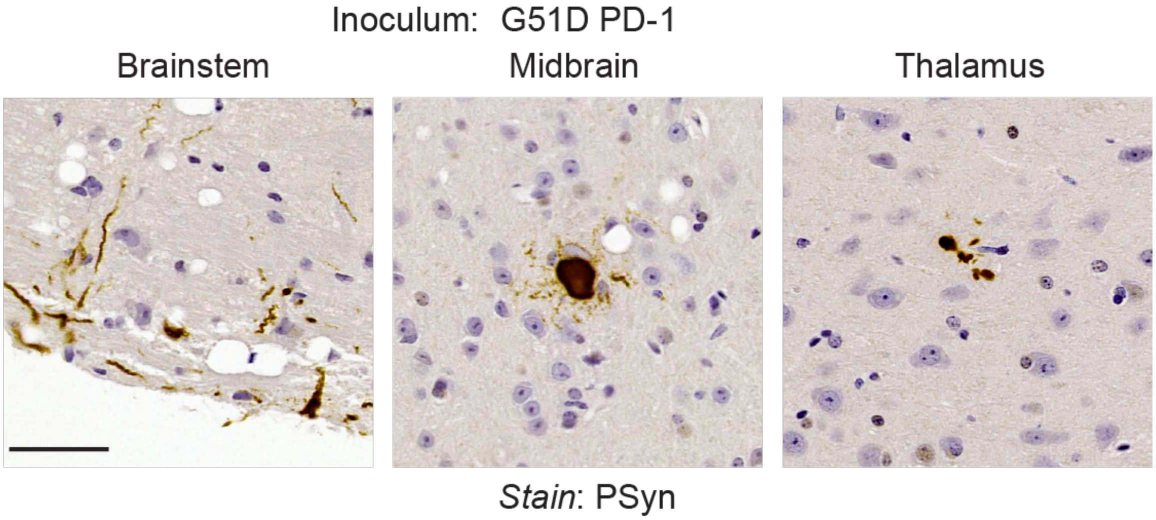

Fig. S4 Other brain regions with PSyn deposits in mice inoculated with G51D PD. Images of PSyn-stained sections (EP1536Y antibody) from the brainstem, midbrain, and thalamus of TgM83$^{+/-}$ mice at 543 DPI with G51D PD-1. Scale bar = 50 μm (applies to all images)

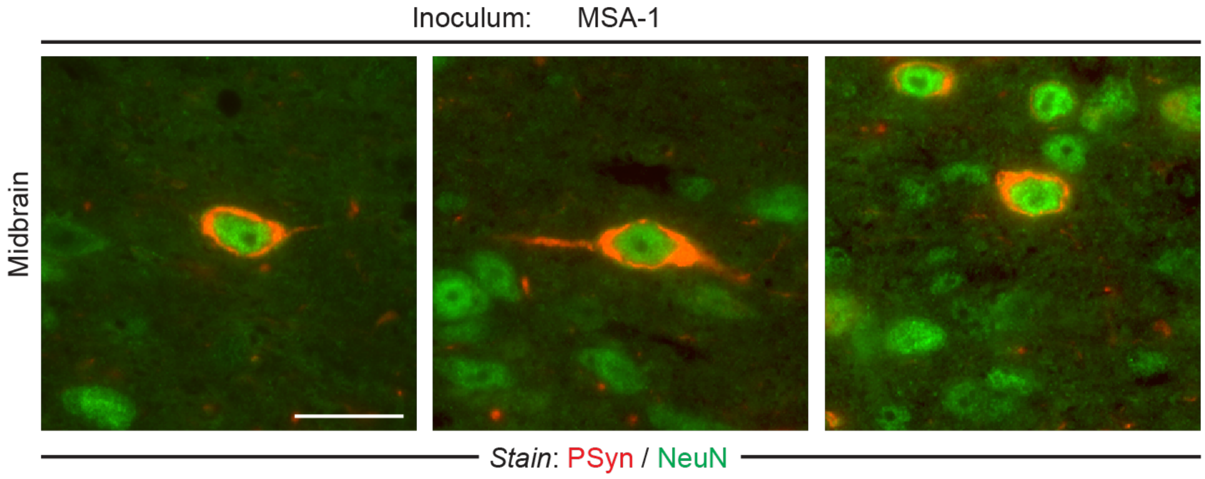

Fig. S5 PSyn deposits in mice inoculated with MSA are present in neurons. Images of sections from the brainstem of a clinically ill TgM83$^{+/-}$ mouse at 257 DPI with the MSA-1 sample stained with antibodies against PSyn (red) and NeuN (green). Scale bar = 20 μm (applies to all images)

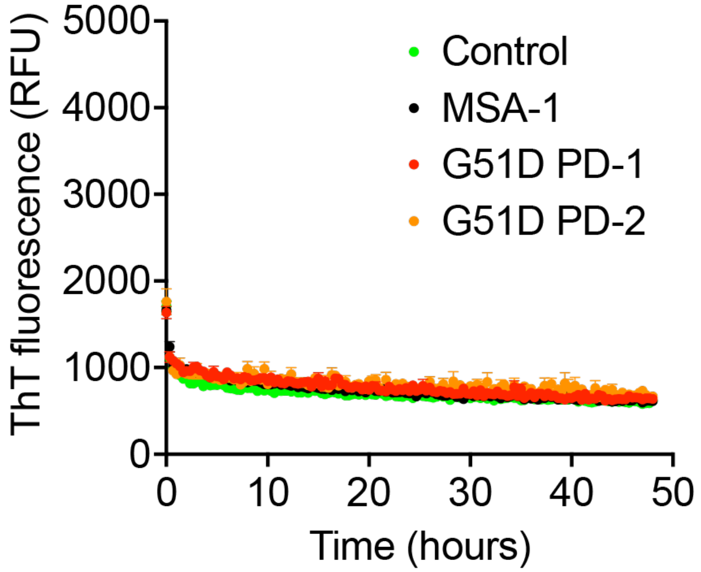

Fig. S6 No amplification of α-syn aggregates in an α-syn SAA with G51D-mutant human α-syn as the substrate. ThT fluorescence curves for α-syn SAA experiments using an MSA-enhanced buffer and recombinant G51D-mutant human α-syn as the substrate. Reactions were seeded with α-syn aggregates from human G51D PD, MSA, or control brain homogenates. Each data point represents the mean ThT fluorescence ± s.e.m. from 4 technical replicates

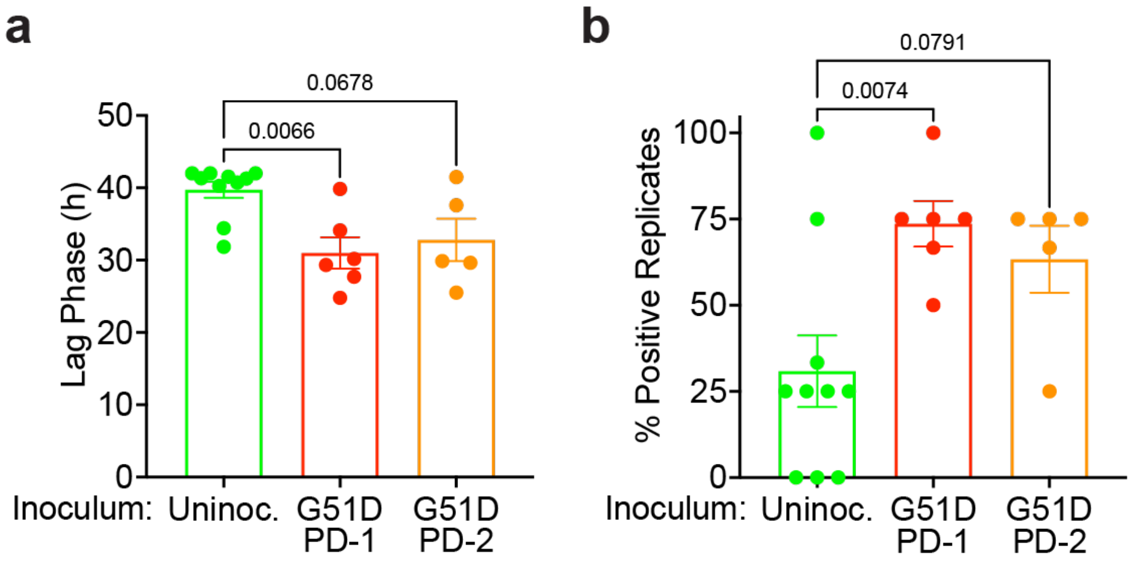

Fig. S7 Kinetics and efficiency of α-syn aggregate amplification in the α-syn SAA. a Lag phases in an α-syn SAA (MSA-enhanced buffer) using brain samples from aged uninoculated TgM83$^{+/-}$ mice (n = 10), G51D PD-1 mice at 543 DPI (n = 6), or G51D PD-2 mice at 540 DPI (n = 5) as seeds. Data is mean ± s.e.m. *P* values were calculated using a Kruskal-Wallis test followed by Dunn's multiple comparisons test. **b** Percentage of technical replicates for brain samples from aged uninoculated TgM83$^{+/-}$ mice (n = 10), G51D PD-1 mice at 543 DPI (n = 6), and G51D PD-2 mice at 540 DPI (n = 5) that were positive in the α-syn SAA. Data is mean ± s.e.m. *P* values were calculated using a Brown-Forsythe ANOVA test followed by Dunnett's T3 multiple comparisons test
